# Dynamic Surface Wetting and Heat Transfer in a Droplet-Particle System of Less Than Unity Size Ratio

**DOI:** 10.3389/fchem.2018.00259

**Published:** 2018-07-02

**Authors:** Subhasish Mitra, Geoffrey Evans

**Affiliations:** Discipline of Chemical Engineering, School of Engineering, Faculty of Engineering and Built Environment, University of Newcastle, Callaghan, NSW, Australia

**Keywords:** droplet-particle interaction, surface wetting, spreading, recoiling, dynamic contact angle, film-boiling, droplet evaporation, VOF-CFD

## Abstract

Dynamic surface wetting of particles in contact with droplet is a complex phenomenon ubiquitously encountered in many multiphase systems of industrial importance. In this study, we address this aspect by investigating impact behavior of a water droplet (diameter = 2.9 ± 0.1 mm) in the Weber number (*We*) range from ~4 to 104 on a stationary spherical brass particle (diameter = 10 mm) with and without heat transfer using a combination of high speed imaging and computational fluid dynamics (CFD) modeling approach. In cold state interactions (20°C), droplet exhibited oscillatory interfacial motion comprising periodic spreading and recoiling motion. Interactions involving heat transfer were studied in film boiling regime (350°C) and two outcomes were noted—droplet rebound and disintegration. A coupled Level Set and Volume of Fluid (VOF) approach based multiphase CFD model was utilized to predict the dynamic spread ratio and transient evolution of droplet shape during the interaction. To capture the complex contact line motion realistically, a continuous time varying profile of experimentally measured dynamic contact angles was used as a wall boundary condition for the cold interactions which provided good agreement with experimentally measured droplet spread ratio. In film boiling regime, droplet spread ratio was correlated to impact Weber number and a power law trend was obtained. Rebound and disintegration outcomes were characterized by the droplet-particle contact time. For simulating interactions in film boiling regime, a constant contact angle in the limit of super-hydrophobic surface was implemented in the CFD model to account for the apparent non-wetting effect due to vapor film formation at the contact area. A sensitivity analysis was performed involving three different contact angle boundary conditions (θ_*s*_ = 150, 160, and 170°) to represent the surface hydrophobicity. CFD model predicted interaction outcomes and droplet spread ratios were in reasonable agreement with the experiment at different impact Weber numbers. Increase in spherical surface heat flux and corresponding rise in droplet temperature at different impact Weber numbers were also quantified which showed an increasing trend up to a critical Weber number for droplet disintegration.

## Introduction

Dynamic wetting of particle surface is an important research aspect in the area of multiphase flows. Ample process applications such as spray coating of tablets in pharmaceutical applications (Hardalupas et al., 1999), vaporization of vacuum gas oil feed droplets in contact with catalyst particles in fluid catalytic cracking unit (Ge and Fan, 2007; Mitra et al., 2013, 2015, 2016, 2017; Nguyen et al., 2015; Banitabaei and Amirfazli, 2017), spray drying (Charalampous and Hardalupas, 2017), thermal cracking of bitumen feed in fluid coking unit, scrubbing of particulate matters from off-gas stream (Mitra et al., 2013, 2015, 2016, 2017) require adequate surface wetting of particles. Dynamic wetting of particle surface resulting from such interactions contribute significantly to the liquid distribution on particle surface, associated heat-mass transport processes, and chemical reactions each of which governs the process performance. Admittedly, a complete theoretical description of the droplet collision process with particles is a difficult problem due to the complex interplay among various hydrodynamic and thermodynamic factors such as deformable interface, three phase contact line motion, heat transfer at solid-liquid interface and evaporation at gas-liquid interface which occurs at different length and time scales.

Central to the surface wetting behavior is the three-phase contact line motion which changes drastically as a consequence of the several outcomes that are possible based on interacting droplet-particle size ratio (Δ) such as deposition, rebound, disintegration (Δ < 1) (Ge and Fan, 2007; Mitra et al., 2013, 2016); capture, penetration, disintegration (Δ> 1) (Mitra et al., 2015), deposition, film formation and disintegration (Δ ~ 1) (Bakshi et al., 2007; Gac and Gradon, 2014; Banitabaei and Amirfazli, 2017; Mitra et al., 2017). For Δ < 1 scenario, upon impact on the solid surface, droplet spreads into a liquid lamella wherein the impact kinetic energy is transformed into the surface energy and the spreading process continues until the kinetic energy is completely dissipated and a maximum spreading state is reached. Following this, the spread-out lamella is retracted by the restoring surface tension force to minimize the surface area and initiates the recoiling phase.

The contact line motion in both spreading and recoiling phase is primarily governed by the competition between the inertia, capillary, viscous, and gravity force. Relative dominance of these forces are often expressed in terms of relevant dimensionless numbers i.e., Reynolds number $\left(Re=\frac{d_{d}v_{d}\rho_{d}}{\mu_{d}}\right)$ as ratio of inertia to viscous force; Weber number $\left(We=\frac{\rho_{d}v_{d}^{2}d_{d}}{\gamma_{\mathrm{lg}}}\right)$ as ratio of inertia to surface tension force; Capillary number $\left(Ca=\frac{\mu_{d}v_{d}}{\gamma_{\mathrm{lg}}}\right)$ as ratio of viscous force to surface tension force and Froude number $\left(Fr=\frac{v_{d}}{\sqrt{gd_{d}}}\right)$ as ratio of inertia to gravity force. From the fundamental research aspect, motion of this three-phase contact line is significant as it governs the contact angle condition on solid surface and directly affects the evolution of interface and wetted contact area. The apparent unsteady contact angle differs significantly from the equilibrium static contact angle or Young's contact angle value and exhibits a hysteresis involving a maximum (dynamic advancing) and minimum (dynamic receding) value.

Previous studies investigated different aspects of this short duration (order of few milliseconds) droplet-particle interaction phenomena utilizing high speed visualization technique. These aspects included droplet deposition behavior on particle surface involving spreading and recoiling phase (Mitra et al., 2013; Malgarinos et al., 2016); rebound behavior in film boiling regime (Ge and Fan, 2007; Mitra et al., 2013, 2016); liquid film coating on particle surface and temporal variation in film thickness (Bakshi et al., 2007; Banitabaei and Amirfazli, 2017; Mitra et al., 2017); and disintegration behavior (Hardalupas et al., 1999; Mitra et al., 2013, 2016, 2017; Charalampous and Hardalupas, 2017).

Alongside experimental studies, numerical modeling involving complete solution of viscous form of the Navier-Stokes equation with moving gas-liquid interface incorporating surface tension force is considered to be a useful tool to gain insights into the complex interaction mechanisms and quantify the wetting behavior and associated heat transfer where applicable. Although significant effort could be noted in the numerical modeling aspect on droplet impact behavior on a flat surface due to its application in spray cooling, fewer studies are indeed available on the droplet impact behavior on particle surface. Of few reported studies, mainly three numerical approaches could be noted—a combined level-set and immersed boundary method (Ge and Fan, 2007), VOF (Mitra et al., 2013, 2015; Malgarinos et al., 2016, 2017a,b); and Lattice Boltzmann Method (Gac and Gradon, 2014).

Ge and Fan (2007) simulated surface wetting behavior of an acetone droplet on a brass particle (Δ < 1) in film boiling regime (200–300°C) in low Weber number range (*We* = 3–20). A thin intervening vapor layer was assumed to exist at the liquid-solid interfacial area which was accounted by a 2D vapor flow model without requiring a contact angle boundary condition. Mitra et al. (2013) utilized an experimentally measured dynamic contact angle profile set over discrete time interval into their CFD model to simulate spreading ratio and droplet shape evolution in cold state interactions (*We* = 8) and a constant contact angle of 180° for film boiling simulations (*We* = 8 and 84, *T*_*p*_ = 250°C). Malgarinos et al. (2016) used a constant static contact angle boundary condition (θ_*s*_ = 90°) in their CFD model with adaptive mesh refining and showed reasonable agreement with the time varying spread ratio for a low Weber number case (*We* = 8) reported in Mitra et al. (2013).

Noting relatively limited effort in quantifying the surface wetting dynamics in a droplet-particle system specifically when heat transfer is involved, objective of the present study was to examine the particle surface wetting behavior at low droplet impact Weber number range using high speed visualization and CFD modeling both in absence and presence of heat transfer. More specifically, aims were to quantify;

1. Role of contact angle boundary condition on the surface wetting dynamics in low Weber number impact regime in absence of any heat transfer and
2. Sensitivity of varying contact angle boundary condition in film boiling regime and effect of impact Weber number on maximum spreading ratio, and heat transfer involving change in droplet temperature and heat flux during impact.

## Experimental

**Figure 1:**
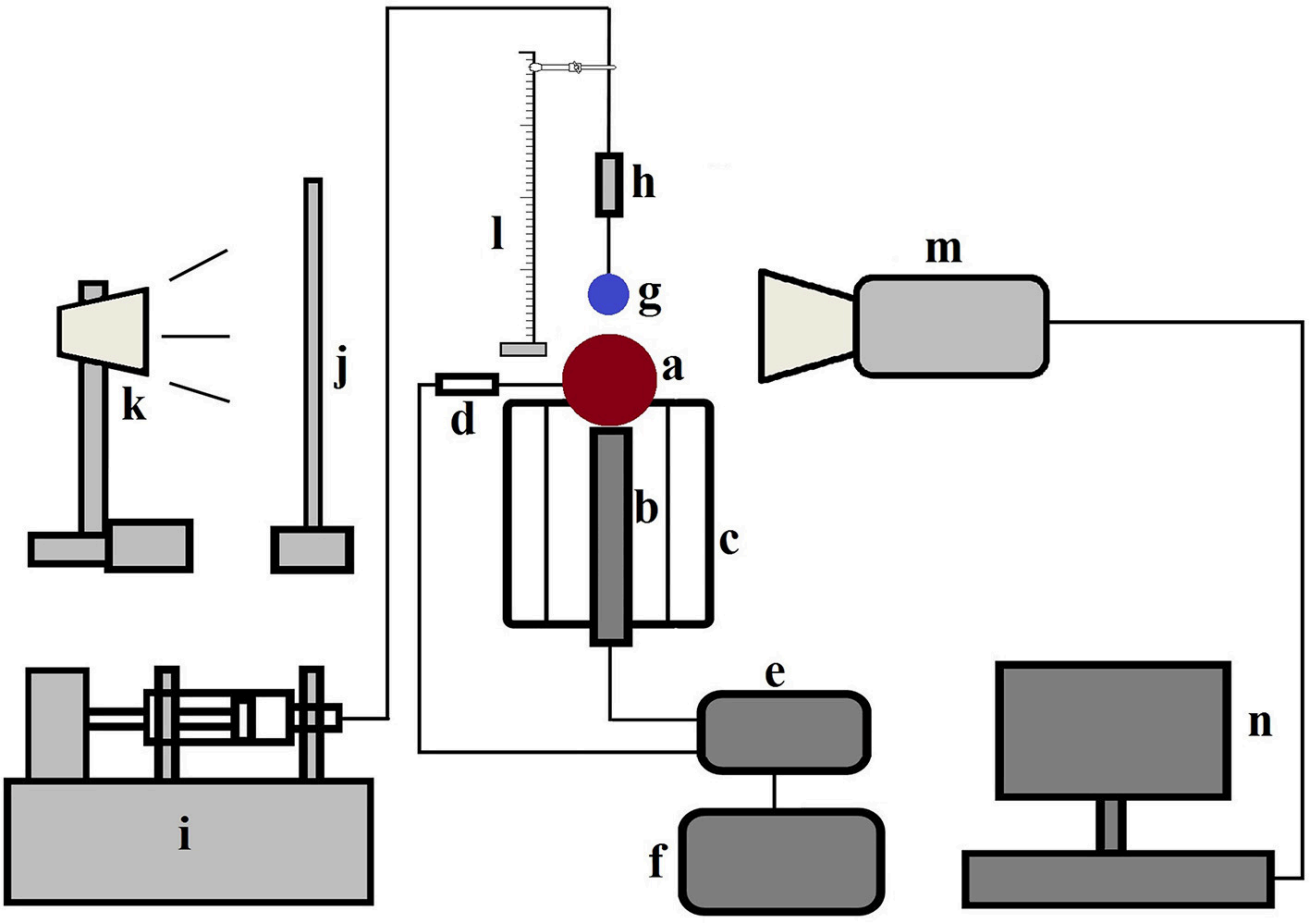
Schematic diagram of the experimental set up—(a) brass particle (b) cartridge heater placed inside the grooved heating billet (c) heating billet with insulation (d) T type thermocouple (e) temperature controller (f) variac with transformer (g) droplet (h) nozzle assembly containing hypodermic needle (i) syringe pump (j) diffuser screen (k) light source (l) height adjustment facility with scale (m) camera (n) computer (Mitra et al., 2016) (With permission from Elsevier).

A schematic of the experimental setup is presented in Figure 1. Experiments were performed using RO filtered water droplets of diameter ~2.9 ± 0.1 mm at both cold state ambient condition (20°C) and film boiling regime (350°C) at different Weber numbers on a 10 mm solid brass sphere. Before each droplet deposition, the sphere surface was carefully cleaned with acetone and allowed for sufficient time to dry. Surface temperature was controlled by a PID controller connected with an embedded T-type thermocouple and a cartridge heater placed in a well-insulated billet. A droplet delivery system with adjustable height (~10–150 mm from the apex point of the sphere) was utilized using a 21G hypodermic nozzle and a precision syringe pump. A single droplet was generated at the nozzle tip at ~2.4 ml/h flow rate by adjusting the pump stroke length which was found to be suitable for deposition purpose. Droplet-sphere interactions were captured using a Phantom v311 high speed camera at 2000 frames per second in shadowgraphy mode using backlighting and a diffuser screen.

An in-house developed MATLAB image processing script was utilized to extract useful data from the captured images. Droplet boundary was marked to separate it from the background and area equivalent diameter was determined. Centroid of the marked droplet was tracked prior to impact to estimate the impingement velocity. Contact angles were determined on both left and right side of the marked interface by computing the inside angle between the two tangents—one drawn to the interface and the other on the spherical surface both passing through the three-phase contact line intersection points (**Figure 3**). Details of the image processing algorithm can be found in Mitra et al. (2016).

## Computational model

### Geometry and mesh

**Figure 2:**
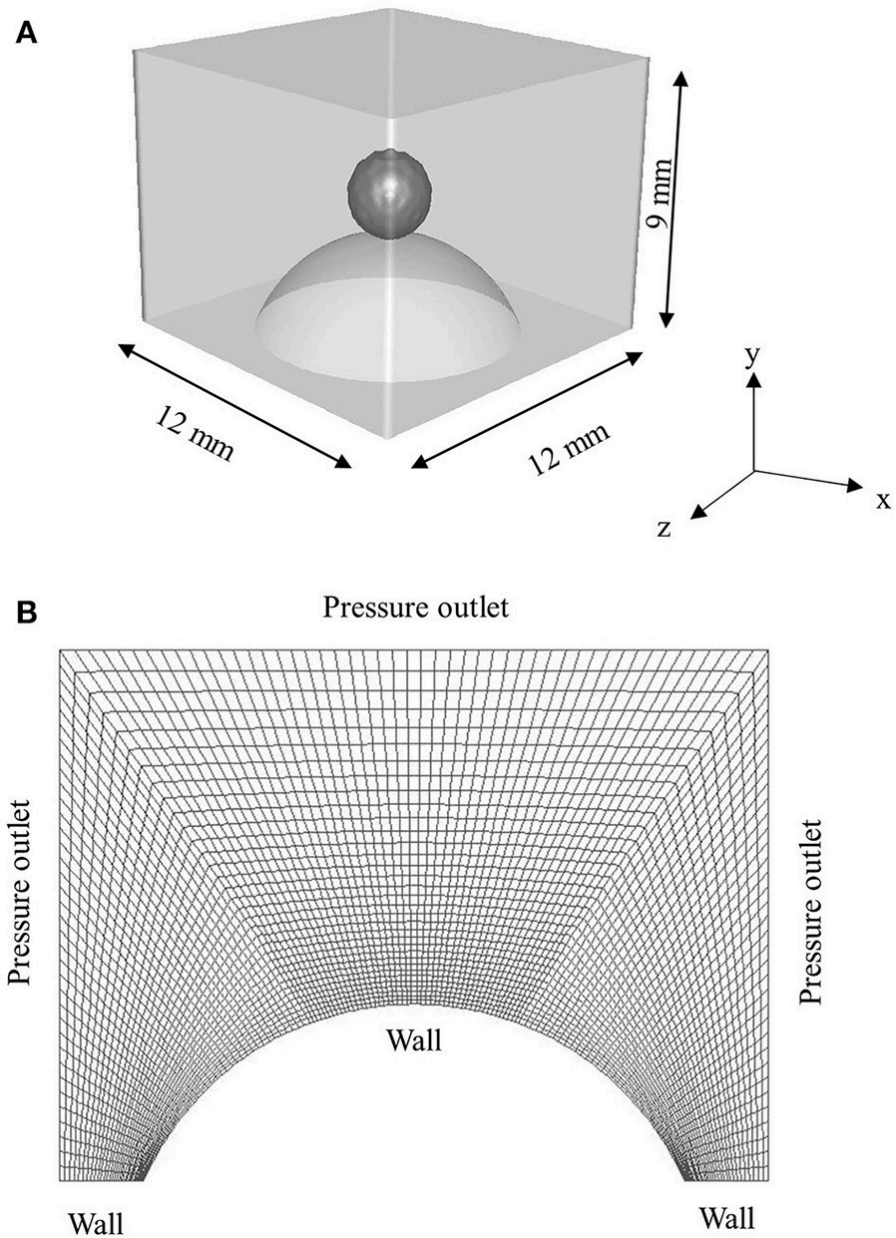
**(A)** 3D Computational domain used for simulation **(B)** sectional view of the hexahedral mesh with boundary conditions.

The computational domain (12 mm × 12 mm × 9 mm) for 3D simulations is presented in Figure 2. Hexahedral meshing tool Ansys ICEM was used to generate the mesh comprising ~0.32 million hexahedral cells. Size of cells were kept lower in the vicinity of spherical surface for better resolution of the three-phase contact line and gradually coarser away from the solid surface. Total 10,592 cells were patched to resolve the droplet. In the present work, total cell number was decided based on a trade-off between a reasonable agreement with the experimental data and computational time which took on average ~2–4 days per case to simulate ~10–20 ms of physical time on a 32 processors workstation.

### Governing equations

A 3D CFD model in Cartesian coordinate system based on the interface capturing coupled level-set and VOF (CLSVOF) (Ansys Fluent theory guide, 2013) approach was implemented in the finite volume method based commercial solver ANSYS Fluent (version: 17). The continuity equation for the liquid phase accounting for the evaporation loss at droplet interface was written as,

(1) $$\frac{\partial \left(\rho_{mix}\alpha_{l}\right)}{\partial t}+\vec{v}.\nabla \left(\rho_{mix}\alpha_{l}\right)=-{\dot{m}}_{evap}$$

where α_*l*_ is the liquid phase volume fraction, ρ_*mix*_ = mixture phase density, *v* is velocity, and ṁ_*evap*_ is the volumetric evaporation rate.

The volume fraction of the continuous phase can be calculated from mass conservation following α_*g*_ + α_*l*_ = 1. The evaporative source term at interface ṁ_*evap*_in Equation (1) was derived as follows:

(2) $${\dot{m}}_{evap}=6\alpha_{l}\left(1-\alpha_{l}\right)\left|\nabla \alpha \right|\left(\frac{dm_{d}}{dt}\right)$$

where |∇α| is interfacial area per unit volume and *dm*_*d*_*/dt* is the evaporative mass flux obtained from Hertz-Knudsen-Schrage kinetic evaporation model as follows (Barrett and Clement, 1992):

(3) $$\frac{dm_{d}}{dt}={\left(\frac{M_{v}}{2\pi R}\right)}^{0.5}\left(\frac{2\chi_{e}}{2-\chi_{e}}\frac{P_{sat}}{T_{l}^{0.5}}-\frac{2\chi_{c}}{2-\chi_{c}}\frac{P_{v}}{T_{v}^{0.5}}\right)$$

where *M*_*v*_ is the vapor molecular weight, *R* is universal gas constant, χ_*e*_ and χ_*c*_ are the evaporation and condensation coefficient, respectively, *P*_*sat*_ is liquid phase saturation vapor pressure corresponding to liquid temperature *T*_*l*_ computed from the Antoine equation and *P*_*v*_ is pressure at vapor side corresponding to temperature *T*_*v*_.

Equation (3) assumes that both incoming (absorption/condensation) and outgoing (reflection/ evaporation) molecular fluxes at droplet interface exhibit Maxwellian distribution behavior which is characterized by the temperature and pressure in the liquid phase near the interface alone. Further assumptions include that molecular transport mechanism depends only on the state variables (pressure and temperature) of the liquid and vapor phase and is independent of the net transfer of mass, momentum, and energy. To depict the probabilistic behavior of molecular interactions with the interface, evaporation coefficient (χ_*e*_) is defined as the ratio of number of molecules transferred to the vapor phase to number of total molecules escaped from the interface. Similarly, condensation coefficient (χ_*c*_) is defined as the ratio of number of the molecules absorbed by the liquid phase to the total number of vapor molecules impinging at the interface. Considering a specific scenario wherein all vapor molecules have the same probability for condensation and both specular (energy of molecules remains conserved) and diffuse reflection (energy of molecules is not conserved) at the interface are possible, under equilibrium assumption, both evaporation and condensation coefficients can be replaced with a single thermal accommodation coefficient for the purpose of simplicity. Nonetheless in real cases, specifically for interface with high curvature, departure from equilibrium state is quite possible which would eventually lead to a lower evaporation rate compared to Equation (3). Accommodation coefficient parameter is strictly determined from experiment and known to have large uncertainty even for a simple molecule like water which varies in the range ~0.01–1.0 (Marek and Straub, 2001). For simulation purpose in the present study, a median value of 0.5 was considered which has been reported in a number of related studies previously (Nikolopoulos et al., 2007; Malgarinos et al., 2017a,b).

A species transport equation was separately solved to account for generation of the vapor phase during evaporation as

(4) $$\begin{array}{l} \left(1-\alpha_{l}\right)\rho_{g}\frac{\partial y_{vap}}{\partial t}+\nabla .[\vec{v}\left(1-\alpha_{l}\right)\rho_{g}y_{vap}]=\\ \;\nabla .[\rho_{g}\left(1-\alpha_{l}\right)D_{vap}\nabla y_{vap}]+{\dot{m}}_{vap} \end{array}$$

where ρ_*g*_ is gas phase density and *y*_*vap*_ is vapor mass fraction.

The momentum equation in addition to pressure, gravity and viscous stress, included a surface tension force ${\vec{F}}_{s}$ to model the interfacial deformation as follows:

(5) $$\begin{array}{l} \frac{\partial (\rho_{mix}\vec{v})}{\partial t}+\nabla .(\rho_{mix}\vec{v}\vec{v})=-\nabla P+\left[\nabla .\mu_{mix}{\left(\nabla \vec{v}+\nabla \vec{v}\right)}^{T}\right]+\\ \;\rho_{mix}\vec{g}+{\vec{F}}_{s} \end{array}$$

where the mixture density and viscosity were calculated based on the volume fraction of each phase as shown below,

(6) $$\rho_{mix}=\alpha_{l}\rho_{l}+\left(1-\alpha_{l}\right)\rho_{g}$$

(7) $$\mu_{mix}=\alpha_{l}\mu_{l}+\left(1-\alpha_{l}\right)\mu_{g}$$

Any additional momentum source term due to evaporation was however not considered in the present modeling framework.

In Equation (5), ${\vec{F}}_{s}$ was modeled according to the continuum surface force model (Brackbill et al., 1992),

(8) $${\vec{F}}_{s}=\sigma_{\mathrm{lg}}\kappa \delta \left(\varphi_{LS}\right)\vec{n}$$

where σ_*lg*_ is surface tension parameter, κ is interface curvature, δ is dirac-delta function and $\vec{n}$ is a unit normal at the interface.

To capture the interface efficiently, a level-set function was used in addition to the phase volume fraction parameter based interface tracking capability of the VOF approach. As the level-set function is smooth and continuous as opposed to the VOF function (discontinuous across the interface), its spatial gradients are computed more accurately. Consequently, accurate estimates of interface curvature and associated surface tension force are obtained. The surface normal at interface was defined as gradient of a level set function ϕ_LS_, and corresponding unit normal was expressed as $\vec{n}=\frac{\nabla \varphi_{LS}}{\left|\nabla \varphi_{LS}\right|}$. Curvature at interface was then written as divergence of the unit normal as $\kappa =\nabla .\left(\frac{\nabla \varphi_{LS}}{\left|\nabla \varphi_{LS}\right|}\right)$.

In Equation (8) and definition of unit normal and interface curvature, ϕ_*LS*_ is a signed function which takes positive value (+ε) in the gas phase, negative value (–ε) in liquid phase, and zero value at the interface and can be written as:

(9) $$\varphi_{LS}(x,y,z,t)=\{\begin{array}{c} +\varepsilon ,ifx,y,z\in \alpha_{g}\\ 0\;at\text{ interface}\\ -\varepsilon ,ifx,y,z\in \alpha_{l} \end{array}.$$

The δ function in Equation (8) ensures that surface tension force in Equation (5) is only computed at the interface and takes zero value elsewhere which was given as

(10) $$\delta (\varphi_{LS})=\{\begin{array}{l} \frac{1+\cos\;\left(\frac{\pi \varphi_{LS}}{a_{l}}\right)}{2a_{l}}\\ 0 \end{array}\begin{array}{c} ,\\ 0 \end{array}\begin{array}{l} for\;\left|\varphi_{LS}\right|\;<\;a_{l\;}where\;a_{l}=1.5h_{gs}\\ for\;\left|\varphi_{LS}\right|\;>\;a_{l} \end{array}$$

where *a* is interface thickness and *h*_*gs*_ is grid spacing.

Transient evolution of both the volume fraction parameter α_*l*_and level set parameter φ_*LS*_ were solved as per the general advection equation (*scalar* ς = α_*l*_, φ_*LS*_) given below:

(11) $$\frac{\partial \varsigma }{\partial t}+\vec{v}.\nabla \varsigma =0$$

It is important to mention that level set function is not mass conserving due to deformation of interface and it needs to be reinitialised at every time step using geometrical interface-front construction method. In this method, both VOF and the LS function values are utilized to reconstruct the interface-front wherein VOF model provides the size of the cut in the cell based on the probable interface location while the direction of the interface is determined by the gradient of the LS function.

Wall adhesion is significant for partial wetting fluids with non-zero contact angle on solid surface. This effect was incorporated in the CFD model expressing unit normal at the wall (${\hat{n}}_{w}$) boundary in terms of unit vectors for fluid and for wall (${\hat{t}}_{w}$) and the contact angle θ as follows,

(12) $$\hat{n}={\hat{n}}_{w}\cos(\theta_{w})+{\hat{t}}_{w}\sin(\theta_{w})$$

Finally, a single energy balance equation was solved for the mixture phase considering that both primary (gas) and secondary phase (liquid) share the same temperature. Additionally, accounting for the phase change source term due to latent heat vaporization, this equation was written as,

(13) $$\begin{array}{l} \frac{\partial }{\partial t}(\rho_{mix}C_{p,mix}T_{mix})+\nabla .(\vec{v}(\rho_{mix}C_{p}T_{mix}+P_{mix}))=\\ \;\nabla .\left(k_{mix}\nabla T\right)-{\dot{m}}_{evap}\lambda \end{array}$$

where *C*_*p, mix*_ and *k*_*mix*_ are mass averaged heat capacity and thermal conductivity of the mixture respectively, and *P*_*mix*_ and *T*_*mix*_ are mixture pressure and temperature and λ is latent heat of vaporization.

The source terms due to phase change in Equations (1), (4), and (13) were implemented through a UDF (user defined function) based on the source term expression given in Equation (2).

### Model parameters and solution procedure

All the thermo-physical properties (density, viscosity, surface tension, heat capacity, and thermal conductivity) of the gas and liquid phase used in the simulations were set as temperature dependent polynomials. No slip boundary condition was applied at the sphere wall in the cold simulation cases. Both static contact angle (θ_*s*_ = 75 ± 3°) and an experimentally measured time dependent continuous dynamic contact angle profile (through user defined function) were used to depict adhesion behavior at solid surface. Pressure outlet boundary condition with zero gauge pressure was applied on all the surrounding faces. For simulating film boiling regime cases, it was assumed that intense vaporization at the solid-liquid interface forms an intervening thin vapor film which renders the surface to be non-wetting and contributes to significant reduction in friction. Without explicitly modeling this vapor layer, a free slip boundary condition (zero shear stress) was rather applied at the solid surface to represent this physical condition (Karl et al., 1996; Mitra et al., 2013; Gumulya et al., 2015). In conjunction with this slip condition, a range of static contact angle boundary condition in the limit of super-hydrophobic surface condition was utilized (see section Dynamic Surface Wetting in Presence of Heat Transfer).

The computational domain was first initialized with zero velocity, pressure, liquid volume fraction, vapor mass fraction and temperature of 293 K. A droplet of diameter 2.9 mm was then patched in the computational domain setting liquid volume fraction equal to 1.0, level-set parameter close to zero and initial velocity equal to the impingement velocity as obtained from the experimental measurement for a particular *We* number case. Governing equations were then solved sequentially in a spatial iteration loop within an outer time loop starting with momentum (Equation 5), mass continuity (Equation 1) with mass loss source term due to evaporation (Equation 2), and correction of velocity and pressure field using a pressure velocity coupling scheme. Energy (Equation 13) was solved next with the energy source term to determine the mixture temperature. Vapor concentration equation (Equation 4) was solved then with the mass source term. Displacement of droplet interface was next obtained by solving the advection equation for phase volume fraction and level set parameter (Equation 11). All user-defined temperature dependent physical properties were updated at the end of the sequence.

For discretization of the momentum and energy equation, a second order upwind scheme was used. Volume fraction parameter and level set parameter were discretised using the Geo-Reconstruct and second order upwind scheme, respectively. PRESTO scheme was used for pressure variable and pressure-velocity coupling required for incompressible flow field was obtained by the SIMPLE algorithm. A residual of 10^−4^ was set for convergence of continuity, momentum, species, volume fraction, and level-set equations while a residual of 10^−6^ was used for the energy equation. All simulations were performed for a duration of 10–20 ms depending on impact Weber number using a time step of 10^−6^ s with 50 iterations per time step. A first order implicit time stepping method was used in all the simulations ensuring global Courant number ~0.1 throughout the simulation.

## Results and discussion

### Dynamic surface wetting at cold state

In the absence of heat transfer, (*T*_*p*_ = 20°C), solid sphere (henceforth referred to as particle) surface wetting behavior was studied at three different water droplet impact velocities. Physical properties of droplet used to report the dimensionless numbers are ρ_*l*_ = 998.2 kg. m^−3^; μ = 0.001 kg.m^−1^.s^−1^, σ_*lg*_ = 0.073 N.m^−1^. Details of the operating conditions are given in Table 1.

**Table 1:** Operating conditions used in the cold state interactions.

**Case**	***d_*d*, 0_* (mm)**	***v_0_* (m.s^−1^)**	***Re***	***We***	***Ca***
1	2.9 ± 0.1	0.32	932	4	0.004
2	2.9 ± 0.1	0.54	1,594	12	0.007
3	2.9 ± 0.1	0.70	2,039	19	0.009

**Figure 3:**
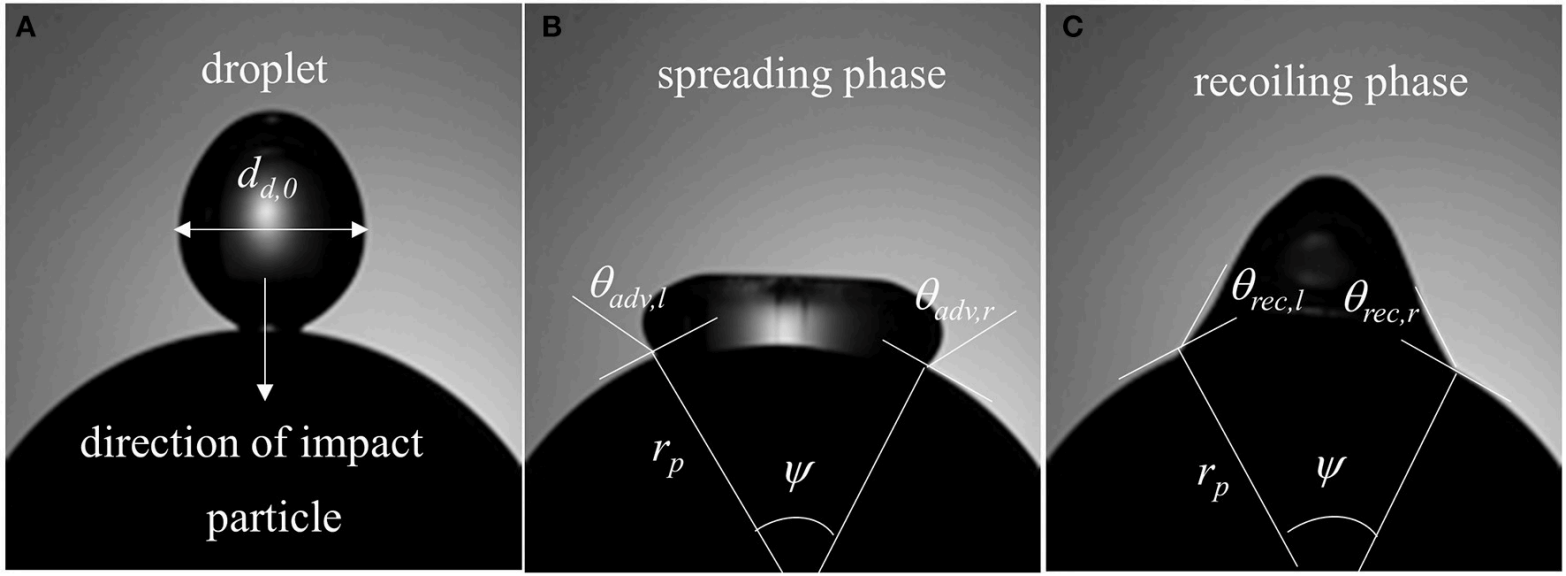
Surface wetting behavior (Case-1*, We* = *4*) and relevant parameters **(A)** droplet at the instant of impact in the direction of gravity **(B)** spreading phase (ψ = central angle, *r*_*p*_ = particle radius, θ_*adv, l*_ = dynamic advancing contact angle at left, θ_*adv, r*_ = dynamic advancing contact angle at right) and **(C)** recoiling phase (θ_*rec, l*_ = dynamic receding contact angle at left, θ_*adv, r*_ = dynamic receding contact angle at right).

**Figure 4:**
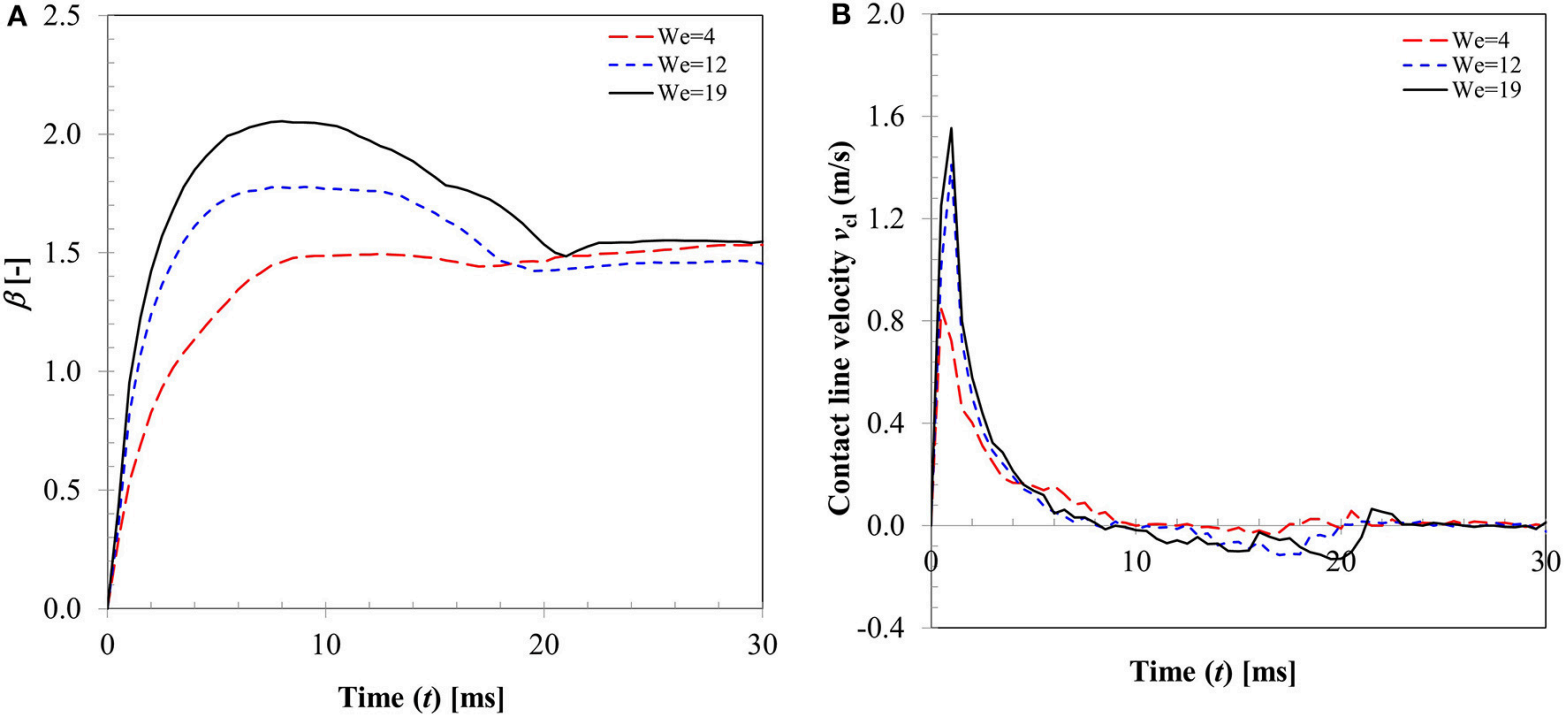
Effect of Weber number (Case 1–3) on **(A)** droplet spread ratio **(B)** contact line velocity.

In a typical low Weber number impact case (case 1–3) below the breakup (disintegration) limit, surface tension and viscous force resist impact inertia which results in deposition outcome (Figure 3). Surface wetting behavior here comprises two distinct phases—spreading and recoiling. An important parameter of interest is the extent of surface wetting which was defined as the spread ratio β (arc length between the two three-phase intersection points) normalized with initial droplet diameter, *d*_*d*, 0_. The wetted perimeter was determined from images by measuring the central angle ψ subtended at the sphere center and sphere radius to give *d*_*d*_ = ψ*r*_*p*_. Effect of Weber numbers on the temporal variations of this non-dimensional wetting parameter is shown in Figure 4A for three different Weber number cases (case: 1–3) in the increasing order. Following impact, due to dominating inertia, in all three cases, droplet quickly reaches the maximum spreading state which is identified by the distinct peaks. The magnitude of the wetting parameter peaks could be noted increasing (~1.5–2.0) with corresponding Weber numbers. Time required to reach the maximum spreading state shows slight left shift in all the three cases which occurs in the time range *t* ~8–10 ms with decreasing order of Weber number. After reaching the maximum spreading state, all three cases exhibit recoiling phase indicated by a sharp decline in the spread ratio parameter which is more prominent in the higher Weber number cases (*We* ~ 12 and 19). This could be explained by the greater rate of conversion of surface energy into kinetic energy in the recoiling phase due to more interfacial area produced at higher Weber number. After the first cycle of spreading and recoiling, few such cycles follow but with much weaker magnitudes due to the dampening effect from competing surface tension and viscous force.

Figure 4B illustrates the temporal variation in contact line velocity obtained in the three representative Weber number cases. Magnitudes of the contact line velocity, as can be noticed, increases with impact Weber number with a slight right shift. The contact line velocity ratios were in the range from ~0.9 to 1.55 which provide an estimation of the maximum possible spreading velocity just after impact. All three velocity profiles pass through a zero cross-over point at *t* ~8–10 ms which denotes the maximum spreading state where momentarily contact line velocity becomes zero (zero kinetic energy). Past this point, contact line velocity profiles exhibit reversal of sign indicating commencement of the recoiling state. The velocity magnitude in the recoiling phase can be noted to be much smaller (−0.1 to −0.15 m/s) compared to spreading phase which indicates weak influence of inertia in the recoiling phase wherein dynamics are primarily controlled by the surface tension, viscosity and gravity force.

**Figure 5:**
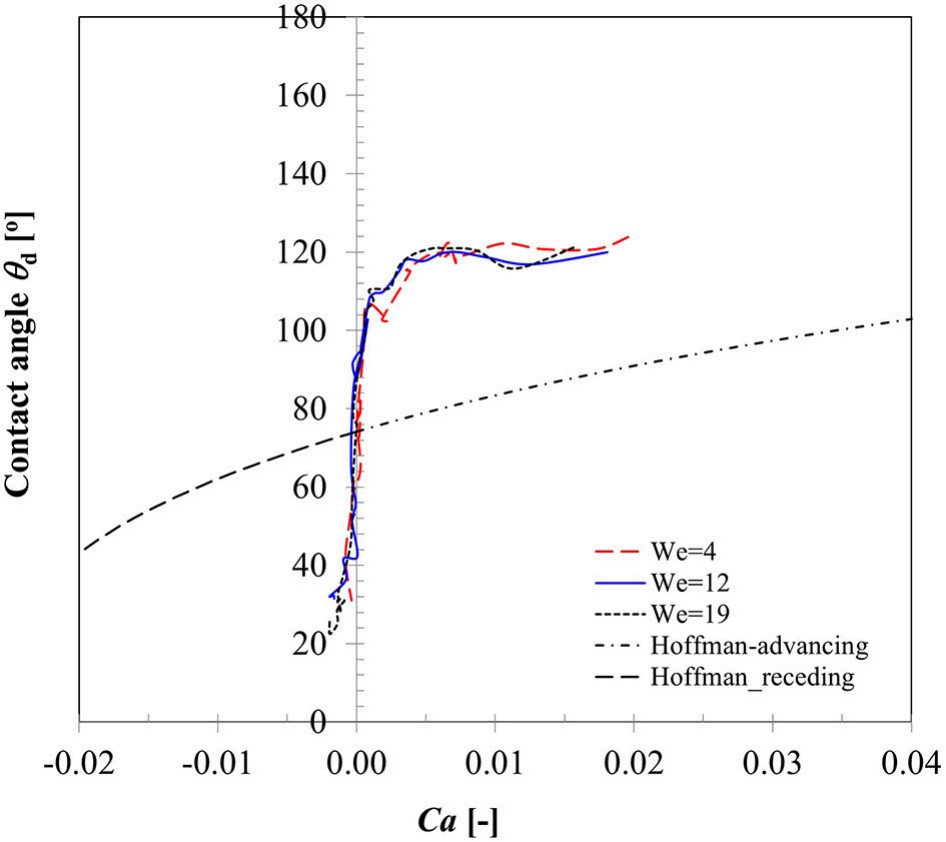
Variation in dynamic contact angle with contact line velocity for Case 1–3 (*We* = 5, 12, 19).

A key parameter in interpreting the surface wetting behavior is the variation in contact angles as the three phase contact line advances or recedes which has been an area of active research for decades (Hoffman, 1975; Dussan, 1979; Kistler, 1993; Hocking, 1995; Ganesan, 2013). Dependency of dynamic contact angles on Capillary number (contact line velocity) for case 1–3 is presented in Figure 5. It could be noted that in the spreading phase due to inertia dominated behavior of contact line motion, contact angle remains almost constant (θ_*d, adv*_ ~ 120°) in all the three Weber number cases. In an earlier CFD modeling study on droplet impact on flat surface, Sikalo et al. (2005) also noted that spreading state is essentially inertia dominated which is indicated by high *Ca* number at the contact line region. Apparent increase in contact angle from the static value at this stage was reasoned to occur due to viscous stress initiating the rolling motion at the advancing gas-liquid interface. A drastic change in the contact angle value is evident near the transition region from spreading to recoiling phase where the contact line velocity passes through a zero cross-over point signifying the maximum spreading state. All contact line dynamics related to droplet impact is located in this transition zone wherein the dynamic contact angle value changes from the advancing to receding mode. Due to weaker velocity magnitude as shown in Figure 4B, dynamic contact angle values were much smaller in the recoiling phase (θ_*d, rec*_ ~ 30°) compared to spreading phase. All the three contact line motion profiles could be seen passing through the zero cross-over point which on the vertical axis reads ~90° and should in principle produce the equilibrium static contact angle. The measured average static contact angle value (θ_*s*_ = 75 ± 3°) however was lower than this value for the same droplet size on brass sphere surface which could be attributed to presence of some hysteresis due to contact line motion.

To compare the prediction capability of an available dynamic contact angle model with these measured values, also plotted here is the well-known Hoffman's (1975) model originally developed for advancing contact angles of a gas-liquid meniscus rising in glass capillary tube for a wide range of Capillary numbers (4 × 10^−5^ ≤ *Ca* ≤ 36). The data was later fitted in Kistler (1993) to produce a useful form widely known as Kistler's law which is given as follows:

(14) $$\theta_{dyn}=f_{Hoff}\left(Ca+f_{Hoff}^{-1}\left(\theta_{s}\right)\right)$$

where *f*_*Hoff*_ and $f_{Hoff}^{-1}$ are the Hoffman and inverse Hoffman function, respectively and written for any independent variable *x* as,

(15) $$f_{Hoff}(x)=\cos^{-1}\left(1-2\tanh\left[5.16{\left(\frac{x}{1+1.31x^{0.99}}\right)}^{0.706}\right]\right).$$

Equation 14 represents a nearly *sigmoidal* (*s* shape curve) indicating dynamic contact angle reaches 180° at very large Capillary number. In Equation (14), using directional sign of contact line velocity (positive and negative) in the definition of capillary number (*Ca*), corresponding dynamic advancing contact angles (θ_*dyn, adv*_), and receding contact angles (θ_*dyn, rec*_) were computed and plotted in Figure 5. Apparently, Equation (14) indicates significant deviations both in the spreading and recoiling phase from the measured values. Sikalo et al. (2005) showed that the existing empirical models for the dynamic contact angles, such as Hoffman's model only provide satisfactory agreement with the experimental data at low capillary numbers (*Ca* < 0.1) and produce significant deviations especially at high capillary numbers. We however note that Hoffman's model significantly under-predicts contact angles even at low Capillary numbers (maximum *Ca* ~ 0.04 in case 1–3) in depicting the three-phase contact line behavior. Similar deviations in the predicted dynamic contact angle values were also noted in the Bracke et al.'s (1989) model (not shown).

**Figure 6:**
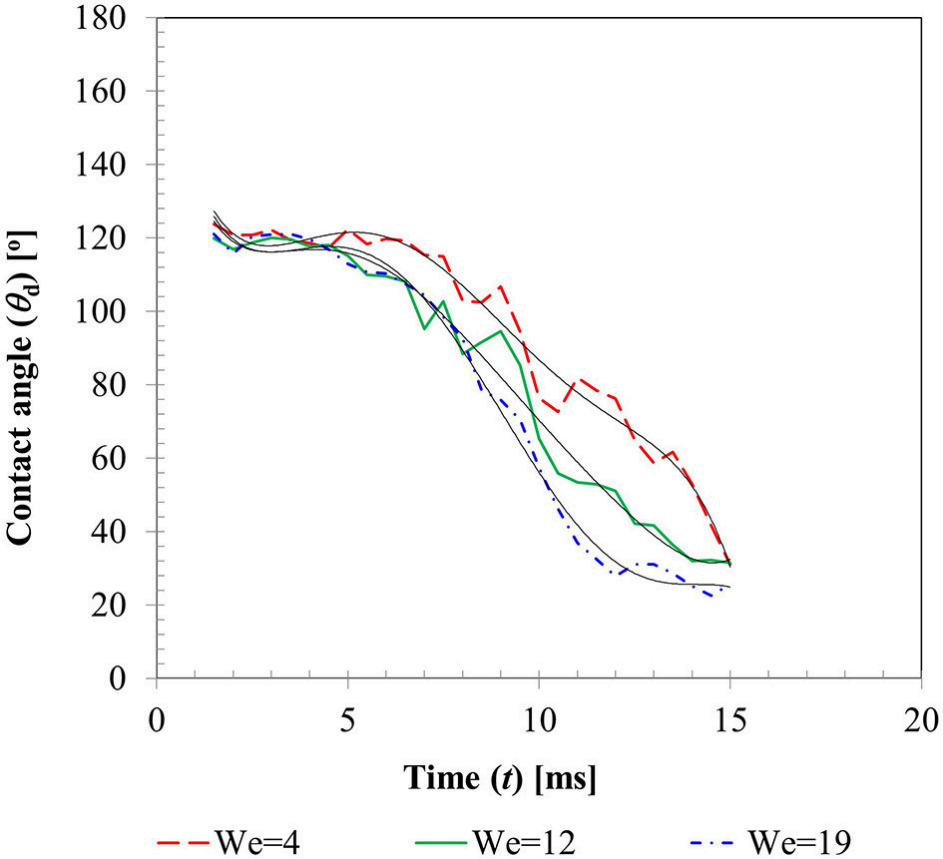
Temporal variation in dynamic contact angle for case 1–3 (*We* = 5, 12, 19).

Figure 6 presents the temporal variations of contact angles (dynamic advancing and receding) obtained for the three different Weber number cases. From the instant of impact (*t* = 0) when droplet and particle are almost at point contact with each other, to *t* ~ 1.0–1.5 ms, contact angles could not be determined due to insufficient resolution of the interface curvature at the liquid-solid contact area. Afterwards, up to *t* ~ 4–5 ms, still in the spreading phase, all the contact angle profiles exhibit a relatively flat regime which can be explained by the relative dominance of inertia over the surface tension force. A steep decline in the contact angle value in the transition zone from advancing to receding phase could be observed in all the three cases with decreasing slopes at higher Weber numbers. This is explained by the larger duration of spreading and recoiling phase observed in higher Weber number cases. Having shown that available empirical models such as Equation (14) do not capture the temporal variations in contact angles well, these experimentally measured contact angle profiles were utilized as wall boundary condition in the CFD model. A sixth order polynomial was fitted to each of these contact angle profiles for interpolating temporal variation in contact angle value in the corresponding time range. Coefficients of these polynomials are provided in Table 2.

**Table 2:** Polynomial coefficients of time varying contact angle profiles.

**Case**	***We***	***a_0_***	***a_1_***	***a_2_***	***a_3_***	***a_4_***	***a_5_***	***a_6_***
1	4	180	−59.428	20.181	−2.923	0.176	−0.003	−4 × 10^−5^
2	12	180	−67.116	26.504	−4.923	0.453	−0.021	4 × 10^−4^
3	19	180	−61.5	21.206	−3.129	0.187	−0.003	−3 × 10^−5^

*Subscript of the coefficients denotes the corresponding exponent of time variable*.

**Figure 7:**
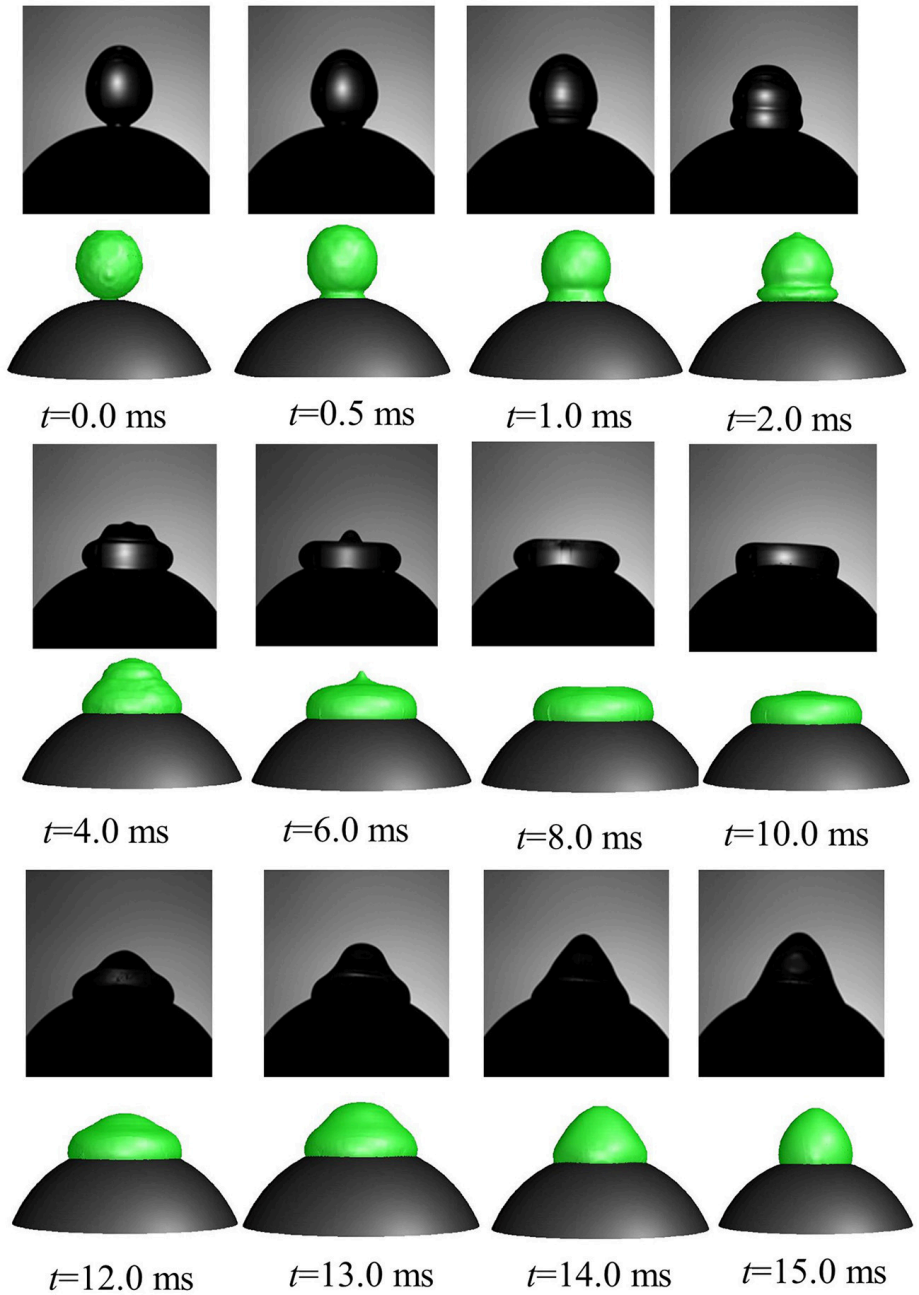
Comparison of CFD model predicted temporal evolution of droplet shape (row 2, 4, 6 in color) with high speed visualizations (row 1, 3, 5 in black and white) (Case 1, *We* = 4).

A comparison of time varying behavior of droplet shape evolution predicted by the CFD model using dynamic contact angle boundary condition and the experimental visualization for *We* = 4 case is presented in Figure 7. A very reasonable agreement was obtained in the predicted dynamics which shows the gradual sinking behavior of the lamella tip into a toroidal shape ring as the droplet spreads and eventually reaches a maximum spreading state (*t* = 8 ms) followed by a gradual increase in the lamella height in the recoiling phase due to retracting action of surface tension force. A definite improvement was achieved using the contact angle profile as a continuous function of time compared to the earlier predicted droplet shape variation presented in Mitra et al. (2013) wherein a discrete time sequence implementation of contact angle boundary condition rendered a relatively coarse interface structure for a similar low Weber number deposition case (*We* = 8).

**Figure 8:**
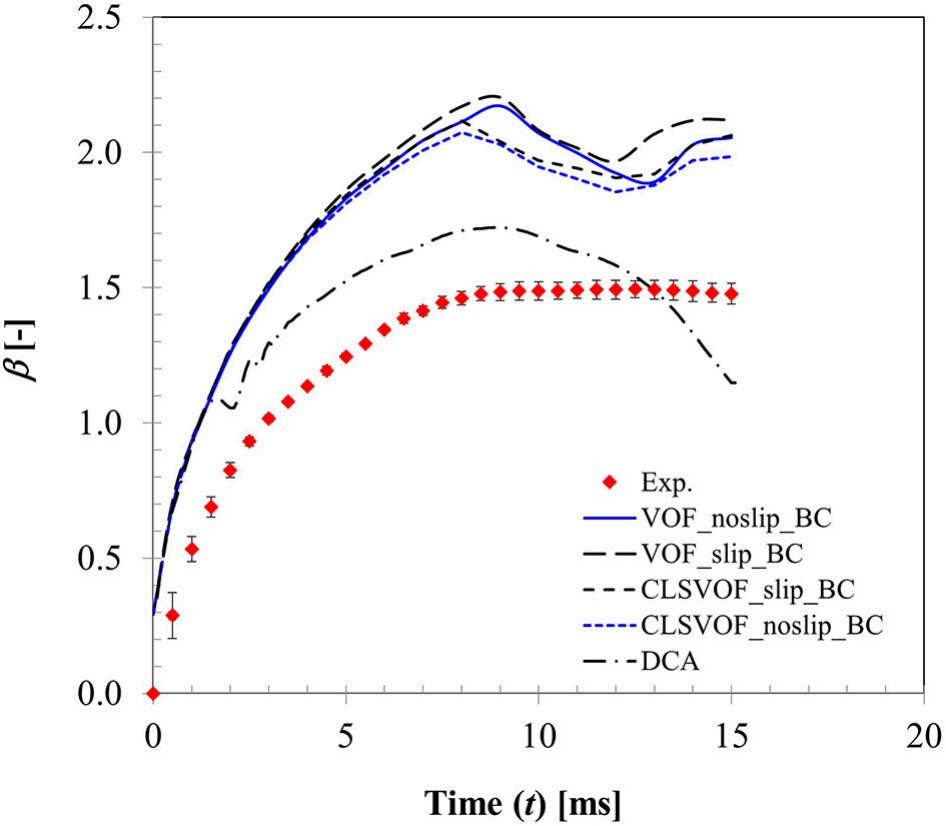
Comparison of CFD model predicted surface wetting behavior based on both static and dynamic contact angle boundary condition (Case 1, *We* = 4).

Figure 8 presents a comparison of the CFD model predicted wetting parameter using both static and dynamic contact angle boundary conditions with the experimental measurement for *We* = 4 case. Also presented here are the effects of the two extremities of slip conditions at particle surface (no-slip and full slip) and interface treatment approach based on VOF and CLSVOF methodology. In CFD computation, wetted surface area hence droplet spread was obtained by integrating the volume fraction of liquid phase in the cells adjacent to particle surface. Clearly, with static contact angle boundary condition, there was almost no difference in the spreading ratio up to *t* ~ 8 ms which covers the spreading phase for all these different combinations. It is quite apparent that even the slip or no-slip boundary conditions do not affect the droplet spreading behavior much where inertia prevails. Past *t* ~ 8 ms, in the recoiling regime, some deviations are apparent in the spread ratio profile which results from different boundary conditions. Between VOF and CLSVOF methodology for interface treatment, deviations in the simulated spread ratio are almost negligible for the no-slip boundary condition however the slip BC condition predicts relatively higher spread ratio as would be expected intuitively due to zero viscous dissipation at the contact line. It was previously noted in Sikalo et al. (2005) that most of the available CFD codes predict droplet shape evolution reasonably well in the spreading phase when droplet motion is primarily controlled by inertia, however they are often not successful in predicting the receding phase which is controlled by the surface tension and surface hydrophobicity.

It is a well-known fact that imposing a no-slip boundary condition on solid surface creates a non-integrable shear stress singularity at the contact line (Huh and Scriven, 1971; Dussan, 1979). To overcome, this issue, often a slip condition (Navier slip condition) is used to capture all the relevant flow and geometry details near the contact line. However, for this purpose, mesh size requires to be of the order of the slip length which is much less than the physical dimension of the droplet but comparable with the intermolecular distance. Any mesh size larger than this slip length leads to mesh size dependent results. Nonetheless using such small slip length entails direct numerical simulation requiring enormous computational resources. In the present study, a VOF based approach was used which utilizes cell face normal velocity in the computation meaning that an implicit slip condition proportional to mesh spacing exists at the no-slip boundary condition near wall (Afkhami et al., 2009). To keep the simulation time reasonable, mesh size near the contact line was kept ~40 μm. Apparently, with this implicit slip condition and the experimentally measured dynamic contact angle profile, a better agreement with the experimentally measured spreading ratio was obtained compared to static contact angle condition without requiring a local mesh adaptation for finer resolution.

A similar observation on the advantage of dynamic contact angle was also noted in Pasandideh-Fard et al. (2001) on their CFD simulation of droplet impact on a flat surface. It was reported that use of dynamic contact angle condition produces more accurate predictions during the spreading and at equilibrium. However, when the contact angle was assumed to be equal to the measured equilibrium value (static contact angle), model predictions were less accurate which over-predicted droplet diameter during recoil. This behavior was also noted in the present study. It is however possible to predict reasonable droplet shape evolution and associated spread ratio with static contact boundary condition alone by local mesh refinement which has been recently demonstrated in Malgarinos et al. (2016).

In previous studies, use of different mesh resolutions has been reported to simulate droplet impact behavior on a solid surface. For example, Nikolopoulos et al. (2007) used ~0.5 million cells (with local mesh refinement) for a geometry size of 15 mm × 15 mm × 10 mm; Malgarinos et al. (2016) used 1.55–2.45 million cells (with 5 levels of local mesh refinement) for a physical geometry size of 60 mm × 30 mm × 30 mm and Gumulya et al. (2015) utilized a 15 mm × 10.85 mm cylindrical boundary involving 1.85 million cells. It is known that interface resolving VOF method bears the computation intensive DNS (direct numerical simulation) characteristic and obtaining truly mesh independent solution is difficult which has dependency on the size of flow structures of interest that need to be resolved. On that note, selection of mesh size in such scenarios is governed by a trade-off between the reasonable model predictions and associated computational cost.

### Dynamic surface wetting in presence of heat transfer

Droplet-particle interactions involving heat transfer was studied in film boiling regime (*T*_*p*_ = 350°C) at ten different droplet impact velocity (see operating conditions in Table 3).

**Table 3:** Operating conditions used in the hot state interactions.

**Case**	***d_*d*, 0_* (mm)**	***v_0_* (m.s^−1^)**	***Re***	***We***	***Ca***
4	2.9 ± 0.1	0.35	1,008	5	0.005
5	2.9 ± 0.1	0.56	1,601	12	0.008
6	2.9 ± 0.1	0.71	2,039	20	0.01
7	2.9 ± 0.1	0.84	2,414	28	0.011
8	2.9 ± 0.1	0.94	2,690	34	0.013
9	2.9 ± 0.1	1.11	3,177	48	0.015
10	2.9 ± 0.1	1.27	3,644	63	0.017
11	2.9 ± 0.1	1.41	4,041	78	0.019
12	2.9 ± 0.1	1.52	4,356	90	0.021
13	2.9 ± 0.1	1.63	4,672	104	0.022

#### Surface wetting parameter

**Figure 9:**
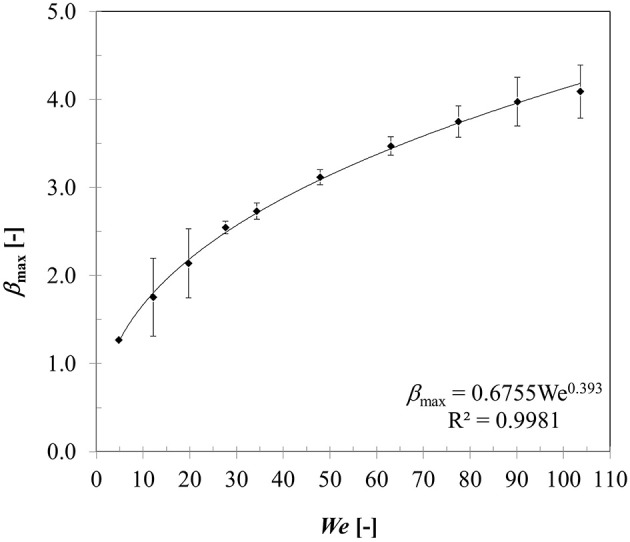
Effect of Weber number on the maximum spread ratio.

Figure 9 presents variation in maximum spread diameter ratio with increasing droplet impact Weber number (5–104) obtained for case 4–13. Due to increase in impact kinetic energy, spread diameter ratio increases leading to increased wetted contact area. A power law trend is quite evident here indicating Weber number dependency of ~0.39. The obtained correlation is very close to Akao et al.'s (1980) correlation $\beta_{\max}=0.61We^{0.39}$(deviation < 15%) obtained from droplet impingement on flat hot metal surface at 400 to 800°C. The empirical Weber number exponent is consistent with the theoretical limit of 0.25 and 0.5 obtained based on a scaling analysis suggested in Clanet et al. (2004) equating kinetic energy to surface energy and disregarding any viscous dissipation on a super-hydrophobic surface.

**Figure 10:**
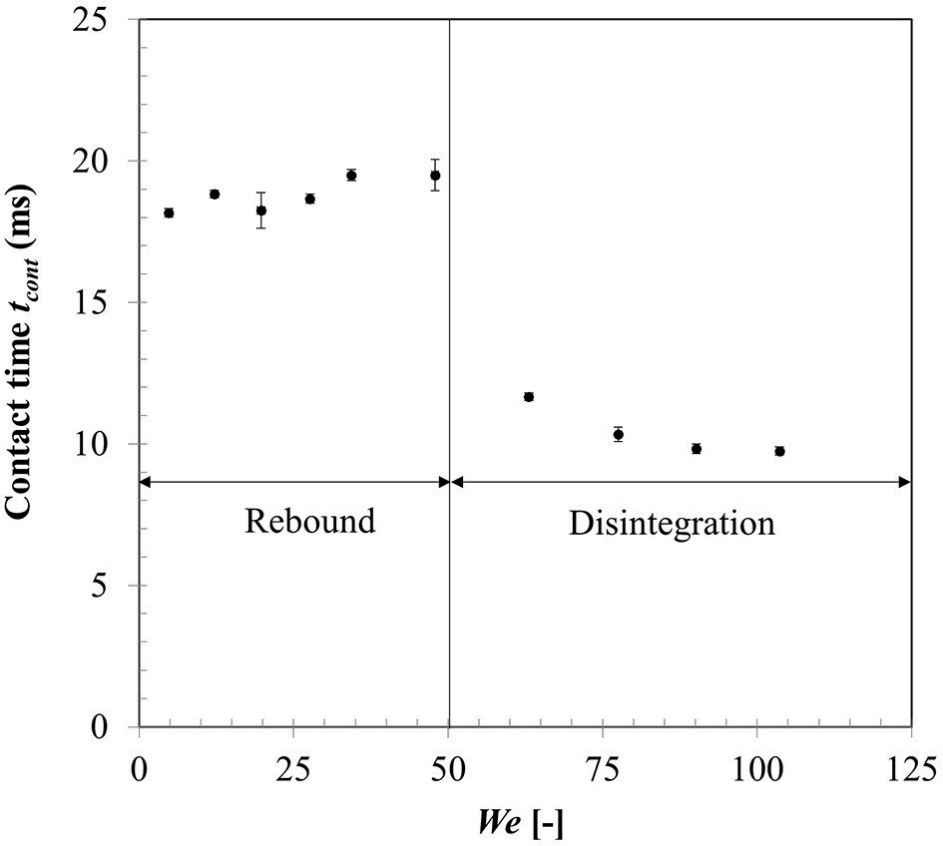
Effect of Weber number on droplet-particle contact time.

Figure 10 shows droplet-particle contact time variation for the same range of impact Weber numbers. Droplet-particle interactions are known to exhibit two outcomes—rebound (droplet bounce off the particle surface) and disintegration (droplet breaks up to produce multiple secondary droplets) in the film boiling regime. These two regimes can be distinctly identified in Figure 10 demarcated by a critical threshold at *We* ~ 50. In rebound regime, droplet exhibits both spreading and recoiling phase however at the end of recoiling phase droplet loses contact with the particle due to intense vapor force at the contact area. Contact times are in the range of ~18–19 ms which are higher compared with the first order droplet vibration time $\tau =\pi \sqrt{\frac{\rho_{l}d_{d}^{3}}{16\sigma_{\mathrm{lg}}}}$ suggested in Wachters and Westerling (1966) which estimates this contact time ~14 ms.

When Weber number is increased past a critical threshold, droplet upon impact continues spreading and liquid mass accumulates in the outer rim. On a non-wetting surface, the larger contact angle creates a greater interface curvature at the periphery of the drop which increases the surface tension force and decreases inertia effect and consequently leads to more mass accumulation at the periphery. Following mass conservation, the connecting lamella on the particle apex decreases in thickness. Eventually due to conduction heat transfer, vapor bubbles are generated within this lamella which erupt and destabilizes the expanding film leading to disintegration outcome (Nikolopoulos et al., 2007; Mitra et al., 2017). Surface tension force then retracts the residual lamella to minimize surface area which leads to ejection of multiple secondary droplets from the rim leading to disintegration. Contact time in the regime drastically reduces due to absence of the recoiling phase. Noticeably, with increasing Weber number, over all contact time decreases however in both regimes separately, contact time remains somewhat independent of the impact Weber number. It was noted that identifying maximum spreading state for a disintegration event at higher impact Weber number had some uncertainties due to side view imaging and a state just before the ejection of secondary droplets from the rim was considered as the maximum spreading state.

The apparent non-wetting behavior in droplet-particle interactions in film boiling regime is attributed to the presence of a thin vapor layer at liquid-solid interface (Harvie and Fletcher, 2001; Ge and Fan, 2007; Mitra et al., 2013, 2016) which renders the surface to appear super-hydrophobic. To investigate effect of this non-wetting behavior on the flow dynamics, a sensitivity study was carried out using different contact angle boundary conditions (θ_*s*_ = 150, 160, 170°) in the limit of super-hydrophobicity.

**Figure 11:**
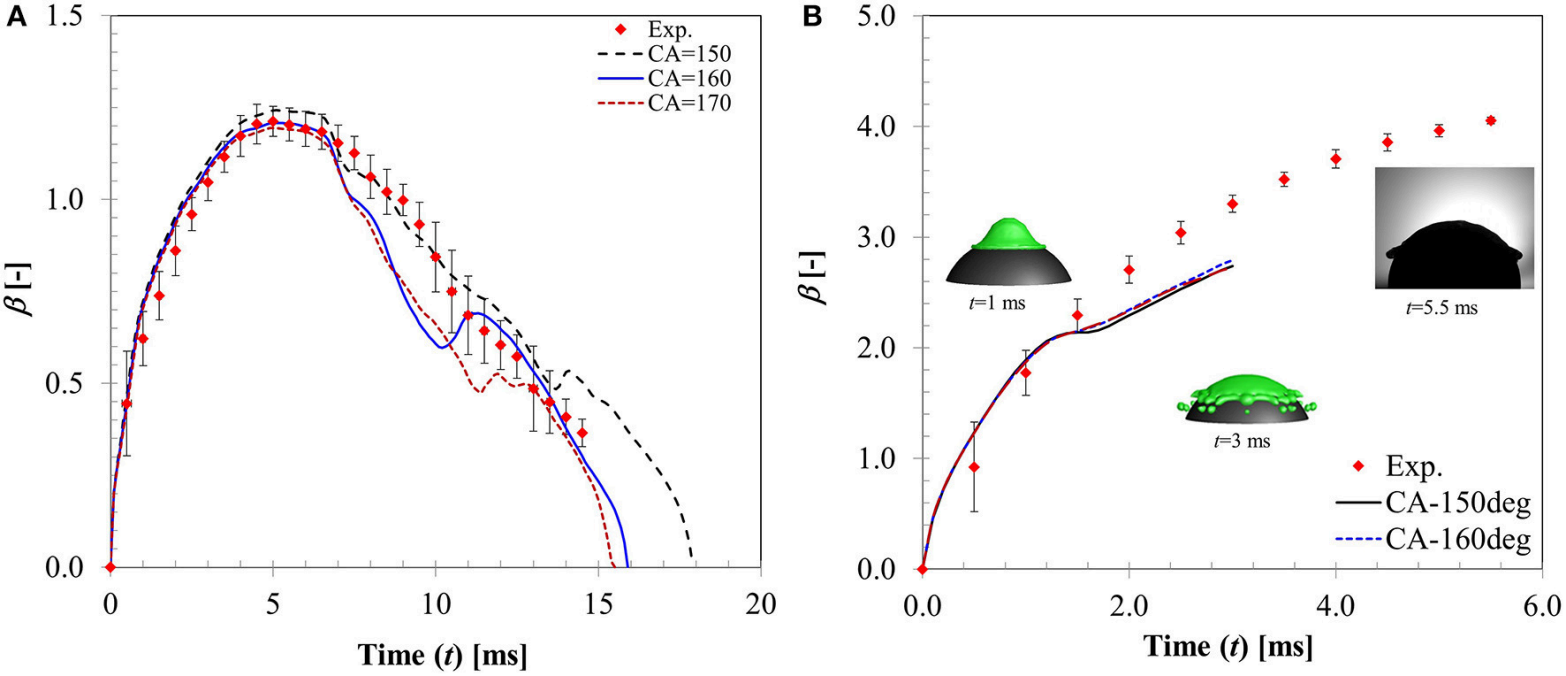
Influence of contact angle boundary condition on the temporal flow dynamics for **(A)** *We* = 5 **(B)** *We* = 104.

In Figure 11, CFD model predicted spread diameter ratio for different contact angle boundary conditions is compared with the experimental measurement for *We* = 5 case. All the contact angle boundary conditions provide reasonable agreement with experimental measurement producing average deviations in the predicted maximum spread ratio as ~9, 13, and 17% for θ_*s*_ = 150, 160, 170°, respectively. Contact times obtained with these boundary conditions are ~18, 16.5, and 15 ms for the three contact angle cases which are also in good agreement with experimental value ~18.3 ms. Clearly, θ_*s*_ = 150° boundary condition provides a better match. Increasing contact angle has an effect on shortening of contact time due to increased surface tension force which is evident from the decreasing contact time trend obtained with increasing contact angle values. This observation is consistent with a contact time value reported in Gumulya et al. (2015) for similar operating conditions (*T*_*p*_ = 250°C, *We* = 24.8) which was 11.5 ms for θ_*s*_ = 180°.

An intermediate Weber number case (*We* = 20, case 6) was also simulated (not shown). Although the model predicted rebound behavior was similar to experiment, deviations in the CFD model prediction were higher (~21% for θ_*s*_ = 150° and ~23% for θ_*s*_ = 170°) specifically toward the maximum spreading and recoiling stage. Both contact angle boundary conditions produce less maximum spread diameter (~1.84 for θ_*s*_ = 150°, ~1.7 for θ_*s*_ = 170° compared with 2.12 in experiment) but the predicted contact times ~22 and 19 ms for θ_*s*_ = 150° and θ_*s*_ = 170°, respectively, were comparable with experiment (~18.3 ms). It is clear from the model predictions that increasing contact angle decreases the maximum spreading ratio but at the same improve contact time prediction due to higher recoiling action.

Figure 11B shows a higher Weber number case (*We* = 104) where disintegration outcome was observed. CFD model also predicts the same outcome however droplet disintegration was predicted earlier. Apparently, all contact angle boundary conditions predict almost identical spread ratio within the time sequence presented with deviations ~15–16% in all cases. CFD simulations indicate that contact angle boundary condition has no observable influence on the flow dynamics in the inertia dominated regime past the critical Weber number for droplet disintegration.

Insignificant effect of contact angle boundary condition on the droplet flow dynamics in non-wetting regime has been identified in few recent studies. Ganesan (2013) compared the effect of four different contact angle boundary conditions—static/equilibrium contact angle, Hocking's model (Hocking, 1995), Jiang et al. (1979), and Bracke et al. (1989) using a finite element based CFD model to simulate droplet impact process. This study demonstrated that contact angle boundary condition does not affect the flow dynamics of droplets on non-wetting surface however for wetting and partially wetting surface, different contact angle models induce different flow dynamics, especially during the recoiling phase. Recently, Banitabaei and Amirfazli (2017) investigated droplet-particle interaction in a Δ ~ 1 system over a range of Weber number (~0.1–1,146) and noted that increasing the contact angle has a considerable effect on geometry of the liquid film and lamella formation, however, increasing the contact angle on a hydrophobic surface beyond a threshold value of 110° did not produce significant effect on the observed lamella geometry.

**Figure 12:**
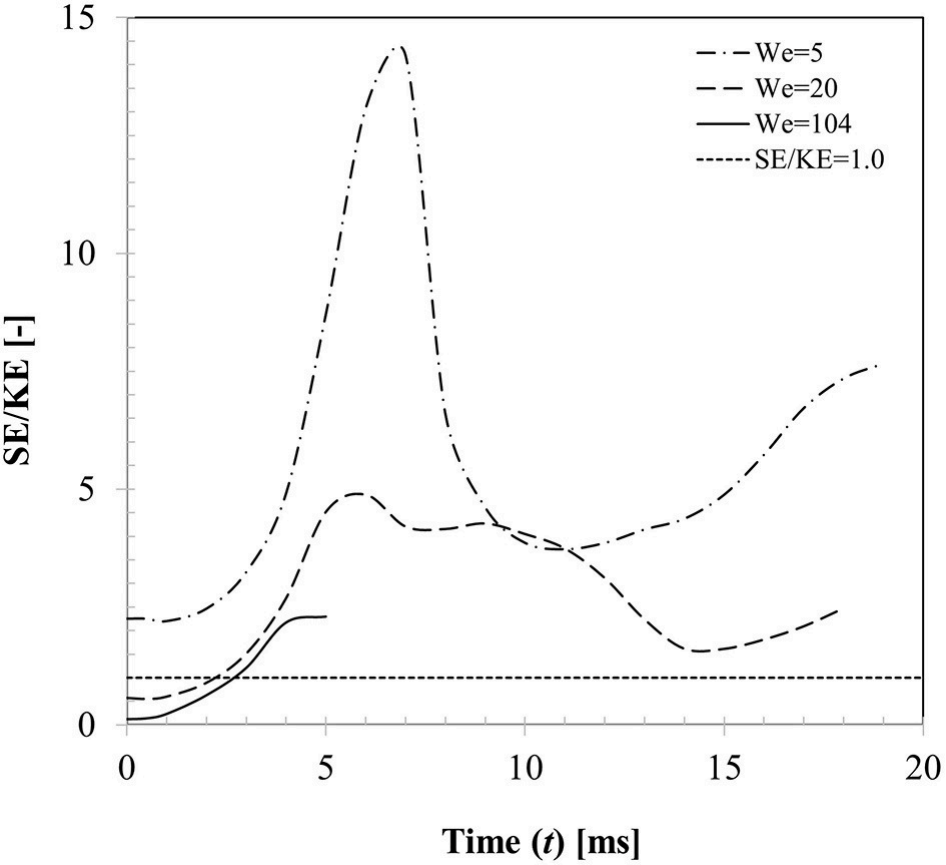
Temporal variation in kinetic and surface energy during interaction at different impact Weber numbers (Case 4, 6, 13, *We* = 5, 20, 104, θ_*s*_ = 150°).

From the energy balance perspective, droplet dynamics is primarily governed by the competition between the kinetic energy ($E_{kin}=\frac{1}{2}m_{d}v_{d,0}^{2}$) and surface energy (*E*_*surf*_ = σ_lg_*A*_int_) considering the fact that energy loss due to viscous dissipation can be ignored on a non-wetting surface. Figure 12 presents the temporal variations of these two energy components for three different Weber number cases (case 4, 6, 13) with respect to a reference line where the surface to kinetic energy ratio is unity. For case 4 (*We* = 5), SE/KE ratio shows rapid increase as the droplet spreads over the particle surface and the ratio reaches a maximum (SE/KE ~14) at *t* ~7 ms which corresponds to the maximum spreading state. This is clearly an unstable state from the energy minimization point of view as surface energy is maximum due to excessive deformation of the interface. Due to this instability, droplet interface is retracted by the surface tension force to minimize the interfacial area and consequently kinetic energy commences to increase from a minimum in the recoiling phase eventually resulting in rebound. It could be noted that throughout the entire dynamics, surface energy always dominates the kinetic energy. For case 5 (*We* = 20), initial kinetic energy of the droplet was higher than its surface energy (SE/KE < 1 at *t* = 0)), however this case also showed rebound outcome indicating maximum spreading ratio at *t* ~6 ms where SE/KE ratio was much lower (~4.9) compared to case 4. For case 13 (*We* = 104), again initial kinetic energy of the droplet was higher than its surface energy, however during spreading phase itself, although surface energy increased, due to dominant inertia and vaporization of connecting lamella, droplet exhibited disintegration outcome.

#### Heat transfer between droplet and particle

**Figure 13:**
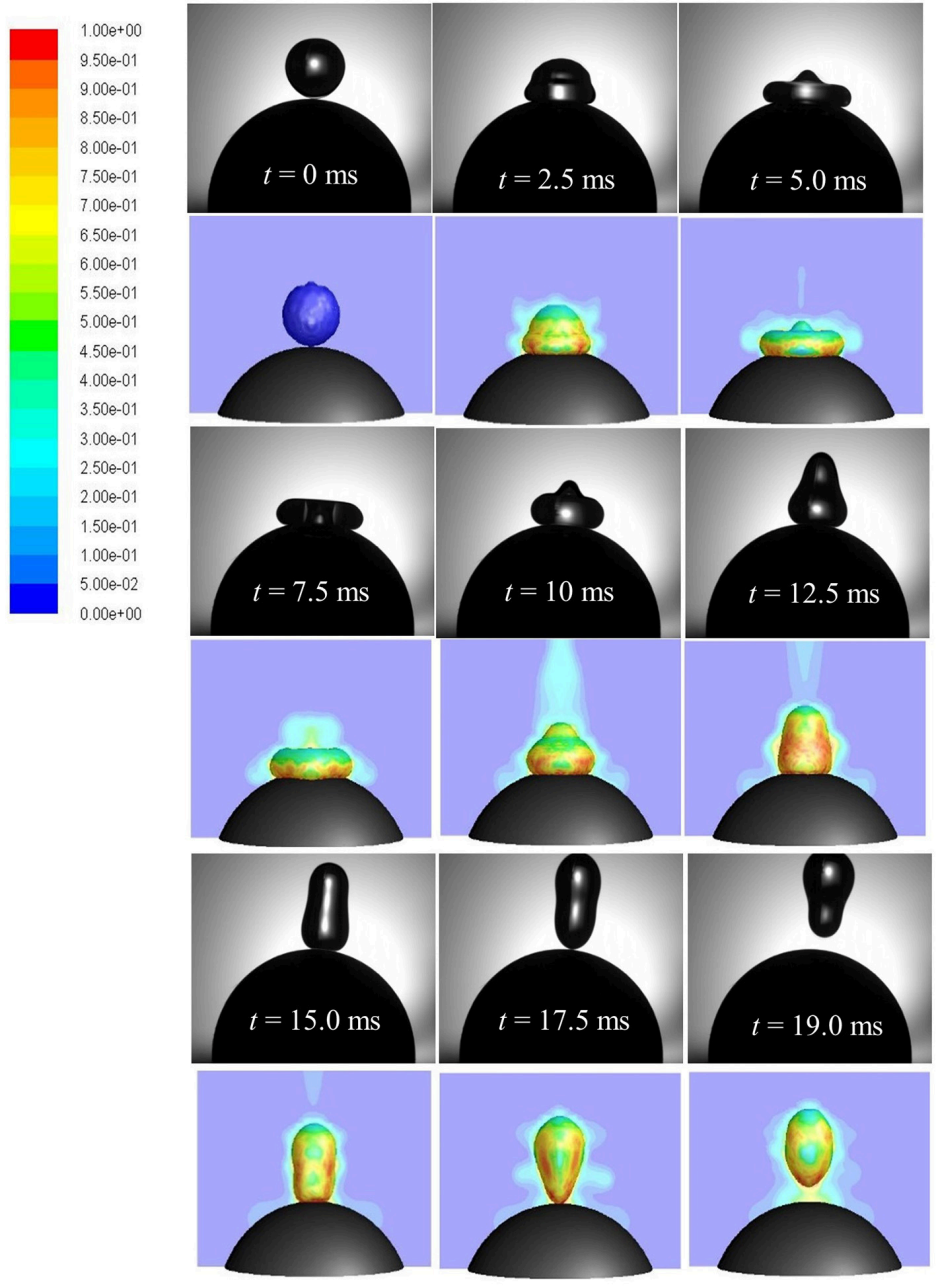
Comparison of CFD model predicted (row 2, 4, 6) droplet rebound behavior with high speed imaging (row 1, 3, 5). Vapor profile around the droplet interface is colored by vapor mass fraction (Case 4, *We* = 5).

A time sequence of droplet shape evolutions for *We* = 5 and corresponding CFD simulations with propagation of vapor field (colored by vapor mass fraction) is presented in Figure 13. Upon impact, droplet spreads in radial direction wherein impact kinetic energy is converted into surface energy and reaches the maximum spreading state at *t* = 5 ms. During the spreading phase, heat is transferred from the solid to the liquid phase. This energy transfer to the droplet increases its mean temperature while liquid vaporizes at the solid-liquid interface due to elevated temperature gradient between droplet and the particle. If the heat transfer rate is large enough during impact, a thin vapor film forms at the solid-liquid interface. The theoretically minimum temperature required for this vapor film to exist (Leidenfrost or minimum film boiling temperature) can be estimated as $T_{Leid}=\frac{27}{32}T_{c}$ (Chandra and Avedisian, 1991) where *T*_*c*_ is the critical temperature. For water *T*_*c*_ = 647 K which estimates the theoretical temperature to be ~545 K (273°C). In the experiment, solid surface temperature was kept 350°C which was well above the minimum film boiling temperature and ensured that film boiling scenario actually occurred.

At the maximum spreading stage, vapor production rate is highest due to formation of maximum wetted contact area. Droplet in this state rests on a thin vapor film (< < droplet radius or wetted contact area) and the produced vapor escapes sideways through this narrow film (order of few microns). In the limit of very low Reynolds number (*Re* < 1), flow and pressure drop can be related using the lubrication theory which equates only the pressure gradient term to the viscous diffusion term in the Navier-Stokes equation (Equation 5) while discarding the effect of other terms. Below the disintegration limit, after the maximum spreading state (wetted area) is reached, vapor force due to pressure drop at the liquid-solid interface becomes maximum which causes the spread-out lamella to retract. Eventually, when the upward acting vapor force due to flow pressure drop in the film is able to overcome the downward acting gravity force on droplet, rebound occurs (*t* = 17.5 ms).

Stability of the vapor layer at the wetted contact area has significant impact on the observed interaction outcome. Burton et al. (2012) showed that smaller diameter Leidenfrost drops having wetted contact area length scale much smaller than the capillary length scale $l_{cap}=\sqrt{\frac{\sigma_{\mathrm{lg}}}{\rho_{l}g}}$ (~2.73 mm for water), have a more stable vapor layer compared to larger size drops where pressure drop in the vapor layer cannot balance the gravity force. In the present study, average droplet diameter was ~2.9 mm and considering the β ratio from the various cases (Figure 9), it could be realized that for wetted contact area length scale (β*d*_*d*, 0_) fluctuates around the capillary length. Due to associated inertia, contact line motion is dynamic which overcomes the droplet gravity in the initial stages of spreading. It could be expected that a uniform vapor layer possibly is never established under the operating conditions used in the present study and fluctuations in the vapor layer thickness are indeed inevitable.

An estimate of this thin film thickness can be obtained from the expression for Leidenfrost drop as $e_{film}={\left(\frac{d_{d,0}}{2}\right)}^{4/3}{\left[\frac{g\mu_{vap}k_{vap}\rho_{l}\left(T_{p}-T_{b}\right)}{\sigma_{\mathrm{lg}}^{2}\lambda \rho_{vap}}\right]}^{1/3}$ suggested in Biance et al. (2003) which gives ~8 μm using water physical properties for liquid and gas phase at ambient condition (20°C) and saturation condition (100°C), respectively. Clearly, resolving such small thickness in the CFD framework leads to a computationally prohibitive multiscale problem. In the earlier work of Harvie and Fletcher (2001) on droplet impact on a heated flat surface, the vapor film was modeled separately based on lubrication approximation as a one-dimensional sub-model outside their 2D CFD code. Later Ge and Fan (2007) extended the approach into two dimensions into their 3D CFD model. The present CFD model includes an evaporation model (Equation 2) which directly accounts for this vapor field however due to limitation in cell resolution at the contact area, the vapor layer contribution could not be directly substantiated.

Also shown here is the evolution of vapor field around the droplet interface on a cross sectional plane colored by the vapor mass fraction which shows a distribution of vapor phase with maximum vapor fraction at the interface and zero (initial vapor fraction in the CFD model was taken as zero) further away from the interface. All heat transfer takes place during the brief period (contact time ~18 ms in rebound regime and ~9 ms in disintegration regime) when the droplet makes a physical contact with the hot particle surface. In absence of any external convection, dominant heat transfer mechanism here is the conduction at solid-liquid interface and internal convection within the droplet. Due to smaller contact duration, droplet temperature increase was small and consequently computed vaporized droplet mass was found to be insignificant (< < 1% in all cases). This is consistent with the earlier results on droplet vaporization under similar operating conditions (~0.16% in Nikolopoulos et al., 2007; < 0.6% in Ge and Fan, 2007; and < 0.1% in Gumulya et al., 2015).

**Figure 14:**
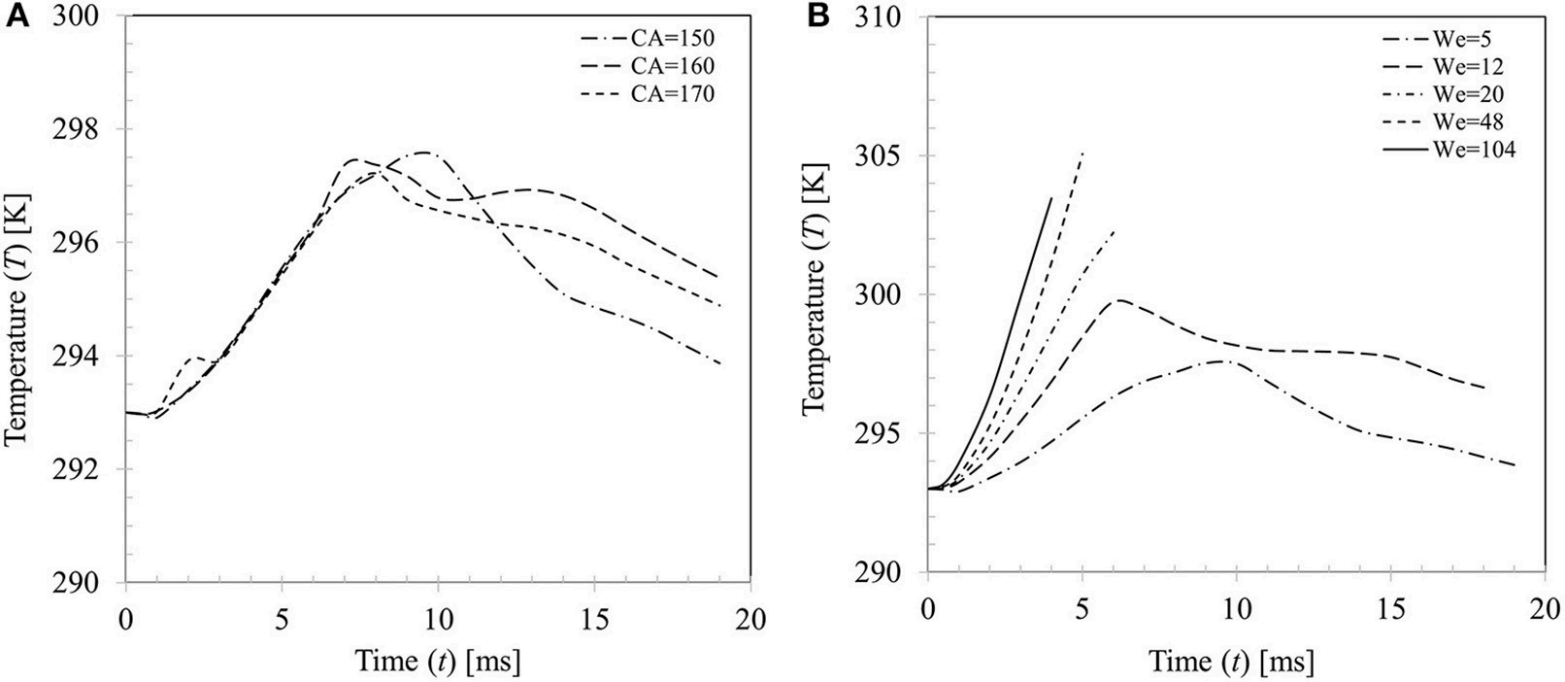
**(A)** Effect of contact angle boundary condition on droplet temperature (*We* = 5). **(B)** Variation in droplet temperature for different impact Weber number cases (*We* = 5, 20, 34, 48, and 104 for θ_*s*_ = 150°).

Change in droplet temperature during impact was computed based on volume averaging as follows $T_{d,avg}=\frac{\underset{V}{\int }\alpha_{d}T_{mix}dV}{\underset{V}{\int }\alpha_{d}dV}$ where α_*d*_ = droplet volume fraction, *T*_*mix*_ = mixture temperature and *V* = domain volume. Figure 14A presents a comparison of the estimated droplet temperature for the three contact angle boundary conditions θ_*s*_ = 150, 160, and 170° for case 4 (*We* = 5). All boundary conditions predict similar temperature rise (~4–5°C) during the spreading phase (*t* ~7–10 ms) and temperature trends almost collapse on each other. In the recoiling phase, due to retraction of droplet hence reduction in wetted contact area, heat input to droplet decreases and at the same time due to evaporative cooling effect, droplet temperature decreases. There are some apparent discrepancies in the predicted droplet temperature in the recoiling phase for different contact angle boundary conditions which vary within ±2°C.

Figure 14B shows variations in the average droplet temperature at different impact Weber numbers. Generally, droplet temperature increases with increase in Weber number up to *We* = 48. This can be explained by the increasing maximum spread ratio hence the wetted contact area for heat transfer at higher Weber number. The discernible peaks in temperature profiles (~298–305 K) indicate the maximum spreading state where wetted area for heat transfer is maximum. A left shift in the observed peaks is apparent at the corresponding Weber numbers which indicates early occurring of the maximum spreading phase. It could however be noted that at the highest Weber number case (*We* = 104), peak temperature *T*_*d*_ ~ 303.5 K is somewhat lower than the *We* = 48 case (*T*_*d*_ ~ 305 K) This could be explained by the fact that even if a larger wetted area is created at higher Weber number, further heat transfer hence increase in temperature is limited by the disintegration process past a critical threshold (*We* ~ 50) which restricts contact time for conduction heat transfer of the droplet on particle surface.

**Figure 15:**
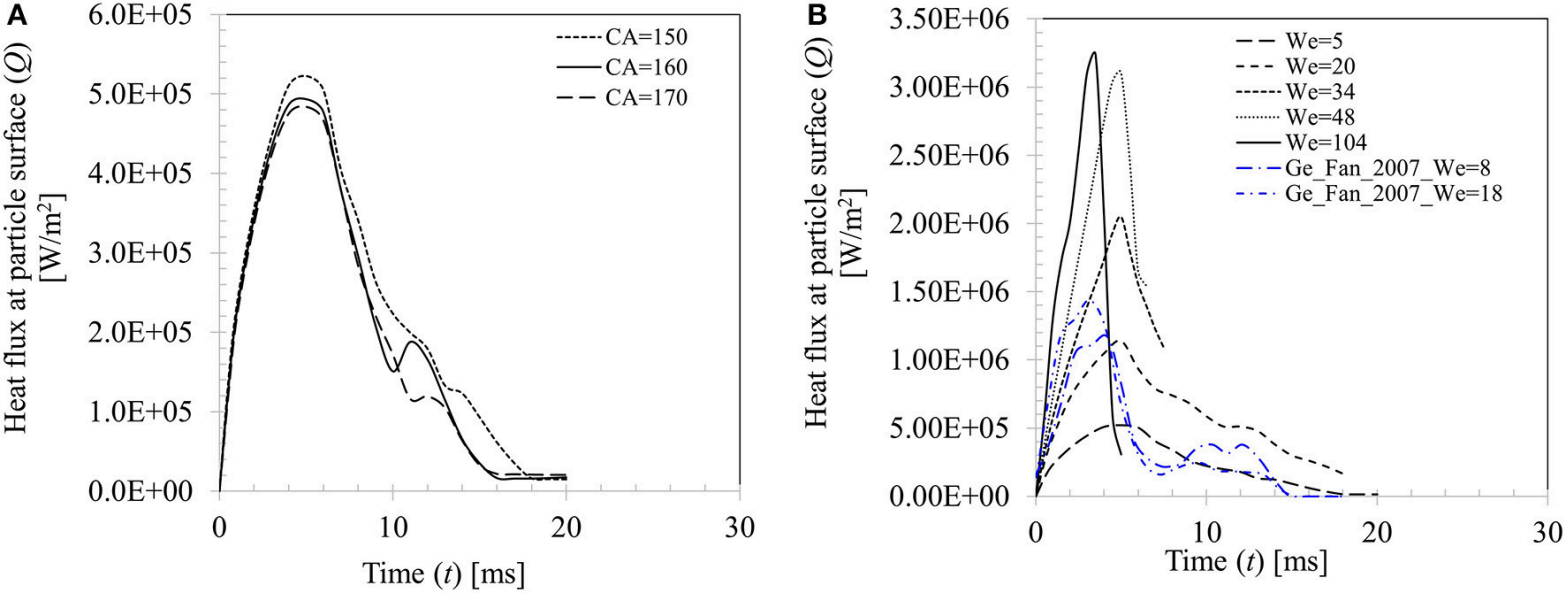
**(A)** Effect of contact angle boundary condition on heat flux for Case 4, *We* = 5. **(B)** Effect of Weber number on heat flux at particle surface (*We* = 5, 20, 34, 48, and 104 for θ_*s*_ = 150°).

Figure 15A presents the effect of contact angle boundary conditions on the computed transient heat flux profile at particle surface for case 4 (*We* = 5). Peak heat flux is slightly higher (5.22 × 10^5^ W.m^−2^) for θ_*s*_ = 150° compared with other two contact angle conditions (4.93 × 10^5^ W.m^−2^ for θ_*s*_ = 160° and 4.84 × 10^5^ W.m^−2^ for θ_*s*_ = 170°) due to decrease in the contact time at higher contact angle boundary condition. Figure 15B shows effect of increasing Weber number on the heat flux profile for θ_*s*_ = 150°. It can be seen that increasing Weber number (*We* = 5–104) leads to increasing heat flux at particle surface which increases by almost an order of magnitude (~5.22 × 10^5^−3.23 × 10^6^ W/m^2^). It could however be noted that after a critical threshold for Weber number is reached (*We* ~ 50), further increase in Weber number, even by doubling it, increase in heat flux is only marginal (~7%). This trend could be explained by the limitation in droplet-particle contact time responsible for the conduction heat transfer duration as explained in Figure 14B. Two more heat flux profiles from the earlier work of Ge and Fan (2007) under similar operating conditions (acetone droplet on brass particle, *d*_*d*, 0_ = 1.8 mm, *d*_*p*_ = 3.2 mm, *T*_*p*_ = 300°C, *We* = 8 and 18) are presented in Figure 15B for a comparison. In these two cases, droplet exhibits rebound outcome and heat flux profiles follow the same trend as obtained in the present study, however peak heat flux in these two cases are comparatively higher for the similar Weber number cases (*We* = 5 and 20) investigated in the present study. This phenomenon could be attributed to the relatively larger droplet-particle size ratio (Δ = 0.56) used in Ge and Fan's (2007) work compared to present study (Δ = 0.29) which led to larger spread ratio hence wetted contact for heat transfer.

A similar observation on the heat flux behavior in the temperature range from *T*_*surf*_ = 50 to 120°C was also noted in Pasandideh-Fard et al. (2001) for droplet impact on a flat heated stainless-steel surface. Increasing impact velocity of droplet was found to marginally increase heat flux from the substrate and the only apparent effect was increase in the wetted contact area over which heat transfer takes place. In another study, using a VOF based CFD model, Nikolopoulos et al. (2007) quantified heat flux for water droplets impinging onto a stainless-steel surface (*T*_*surf*_ = 180°C) which was ~3 × 10^7^ W/m^2^. The reported heat flux value in their work was higher by an order of magnitude compared with the present study. The discrepancy could be attributed to the lower surface temperature used in their work, *T*_*surf*_ = 180^o^C which defines a nucleate to film boiling transition regime. In this regime, surface was moderately hydrophobic which was characterized by a contact angle value of 100°. In absence of an insulating vapor film at the contact area, direct droplet-surface contact was inevitable which resulted in comparatively higher heat flux.

## Conclusion

In the present study, surface wetting behavior of a spherical particle with and without heat transfer was reported at different droplet impact Weber numbers in the range from 4 to 104. In absence of heat transfer, it was shown that implementation of a continuous time varying measured dynamic contact angle boundary condition provides better agreement to spread ratio. It also produces better interface shape evolution behavior specifically in the recoiling phase when compared with the discrete time implementation of contact angle boundary condition earlier reported in Mitra et al. (2013).

Effect of heat transfer on the droplet-particle interaction was studied in the film boiling regime. Maximum droplet spread ratio was correlated to Weber number which exhibited a power law trend. Two distinct outcomes were observed—rebound and disintegration based on droplet-particle contact time and were clearly demarcated by a critical Weber number ~50. Droplet-particle contact time in the rebound regime decreased almost by half in the disintegration regime due to absence of the recoiling phase. Flow dynamics in the film boiling regime was studied for different surface hydrophobicity using three contact angle boundary conditions in the limit of super-hydrophobicity (θ_*s*_ = 150, 160, 170°) to account for an intervening vapor film at the liquid-solid interface. In general, contact angle boundary condition appears to have less influence on the flow dynamics specifically in the spreading phase, which was confirmed by the relative invariance of maximum spread ratio, droplet temperature and heat flux parameter for all three contact angle conditions. Heat flux and droplet temperature increased with increasing Weber number due to creation of larger spread ratio (wetted contact). Droplet temperature rise was predicted to be in the range from ~4 to 12°C while heat flux increased by almost an order of magnitude. Although increasing droplet impact velocity (Weber number) augments heat transfer on particle surface, maximum possible heat flux was shown to be largely limited by the contact time which cannot be increased by increasing impact velocity beyond the critical Weber number threshold for droplet disintegration.

Summarizing, this study aimed to analyse the particle surface wetting phenomenon based on its impact dynamics which is critical for a number of multiphase applications of industrial importance. The interface resolved computational model coupling hydrodynamics with the physics of surface tension, contact angle and heat and mass transfer process provides advanced level of details such as wetted contact area, maximum possible heat transfer, evaporation rate and corresponding variation in droplet temperature. The model is also capable of predicting uneven temperature distribution within the particle body (although not shown) as a result of localized transient surface wetting. All this information is essential to gain insight into the complex phase interactions closely associated with process applications such as spray coating, spray drying, fluid coking, and fluid catalytic cracking and cannot be solely obtained from experiment. Further studies in this area will focus on the collision induced chemical reactions that occur during transient heat transfer at particle surface and has relevance specifically to the fluid catalytic cracking process.

## Ethics statement

This manuscript has not been previously published or is not currently under consideration for publication elsewhere, either in whole or in part.

## Author contributions

SM conducted the experiment, performed CFD modeling, analyzed data and drafted the manuscript. GE provided valuable inputs to the work through insightful discussions and revised the manuscript.

### Conflict of interest statement

The authors declare that the research was conducted in the absence of any commercial or financial relationships that could be construed as a potential conflict of interest.

## Nomenclature

*A* area (m^2^)

*a* interfacial thickness (m)

*C*_p_heat capacity (J.kg^–1^.K^–1^)

*d* diameter (m)

*F*_s_ surface tension force (N/m^3^)

*g* gravitational constant (m.s^–2^)

*h*_GS_ grid spacing (m)

*k* thermal conductivity (W.m^–1^.K^–1^)

*l* length scale (m)

M molecular weight (kg.kmol^–1^)

*m* mass kg

*ṁ* volumetric evaporation rate (kg.m^–3^.s^–1^)

*n* unit normal (-)

*P* pressure (Pa)

Q heat flux (W/m^2^)

*r* radius (m)

*R* universal gas constant (J. kmol^–1^.K^–1^)

*T* temperature (K)

*t* time (s)

*t*^*^ non-dimensional time (-)

*V* volume (m^3^)

*v* velocity (m.s^–1^)

*y* species mass fraction (-)

**Dimensionless numbers**

*Ca* Capillary number

*Fr* Froude number

*Re* Reynolds number

*We* Weber number

**Greek letters**

α dispersed phase volume fraction (-)

β droplet spread ratio (-)

δ Dirac delta function (-)

Δ droplet particle size ratio (-)

ε level set function value (-)

ζ scaler variable (-)

θ contact angle (°)

κ interface curvature (m^–1^)

λ latent heat of vaporization (J.kg^–1^)

μ viscosity (Kg.m^–1^.s^–1^)

ρ density (kg.m^–3^)

σ surface tension (N.m^–1^)

τ characteristic time scale (s)

ϕ level set function (-)

χ accommodation coefficient (-)

ψ central angle (°)

**Super/Subscript**

*adv* advancing

*avg* average

*c* condensation

*cap* capillary

*cl* contact line

*d* droplet

*e* evaporation

*g* gas

*hoff* Hoffman function

0 initial state

*int* interface

*kin* kinetic

*l* liquid

*LS* level-set

max maximum

*mix* mixture

*p* particle

*rec* receding

*s* static

*sat* saturation

*surf* surface

*vap* vapor

*w* wall
